# Novel Polyaniline–Silver–Sulfur Nanotube Composite as Cathode Material for Lithium–Sulfur Battery

**DOI:** 10.3390/ma14216440

**Published:** 2021-10-27

**Authors:** Jing Wang, Ri-Wei Xu, Cheng-Zhong Wang, Jin-Ping Xiong

**Affiliations:** 1 Beijing Key Laboratory of Electrochemical Process and Technology for Materials, Beijing University of Chemical Technology, Beijing 100029, China; xurw@mail.buct.edu.cn (R.-W.X.); czwang@mail.buct.edu.cn (C.-Z.W.); 2 College of Ecology and Resources Engineering, Wuyi University, Wuyishan 354300, China

**Keywords:** lithium–sulfur batteries, polyaniline, silver, cathode materials

## Abstract

The preparation and characterization of a polyaniline–silver–sulfur nanotube composite were reported in this paper. The polyaniline–silver nanotube composite was synthesized via an oxidation-reduction method in the sodium dodecyl sulfate (SDS) solution. After being vulcanized, the polyaniline–silver–sulfur (Poly (AN–Ag–S)) nanotube composite was prepared as active cathode material and assembled into lithium–sulfur (Li–S) batteries with electrolyte and negative electrode materials. When the feed ratio of raw materials (aniline and AgNO3) was 2:1, the initial specific capacity of poly (AN–Ag–S) composite cells reached 1114 mAh/g. The specific capacity was kept at 573 mAh/g, and the capacity retention rate stayed above 51% after 100 cycles. The introduction of Ag into the composite cathode material can effectively solve the poor conductivity of sulfur and improve the Li–S battery performance.

## 1. Introduction

Polyaniline (PANI) has become the key research point in secondary battery cathode materials due to its reversible electrochemical redox capability [1]. After being blended with an appropriate amount of metal nanoparticles, PANI obtains better conductivity and electrical activity, because the existence of metal nanoparticles will offer larger specific surface area, which can adsorb the intermediates during the charge–discharge process, so that the battery performance can be greatly improved [2,3,4,5]. At the same time, the metal nanocomposite structure can solve the problem of electron/hole recombination and stop the re-stacking in other materials, which has great potential application in most other aspects [6,7]. Among all the metal materials, silver (Ag) shows the best conductivity and thermal stability [8]. Therefore, it has great value during the exploration of the PANI–Ag composite, especially in the application of secondary battery.

K. S. Kim et al. [9] successively deposited nano Ag and PANI on graphite fiber and studied the capacitance of the composite via the cyclic voltammetry (CV) method and constant current charge–discharge method. It was found that the composite had the specific capacitance of 212 F/g, better than the pure PANI had. It showed that this composite can be used as electrode material for the super capacitor. M. Sawangphruk et al. [10] studied the capacitance of a porous nano-PANI–Ag composite which was prepared via potentiostatic deposition. It was found that this composite had high specific capacitance (430 F/g) and excellent stability. After 200 cycles, the specific capacitance was 94% of the initial specific capacitance. Therefore, it can be used as a potential electrode material for super capacitors.

In this experiment, the preparation of a polyaniline–silver–sulfur nanotube composite was reported. The polyaniline–silver nanotube composite was synthesized via an oxidation-reduction method in the sodium dodecyl sulfate (SDS) solution. The composite was characterized by FT-IR spectra (IR), X-Ray Diffraction (XRD), Raman spectra, Scanning Electron Microscopy (SEM), Thermogravimetric analysis (TGA), etc. After being vulcanized, the polyaniline–silver–sulfur (Poly (AN–Ag–S)) nanotube composite was further successfully utilized as the cathode material in Li–S batteries. When the feed ratio of raw materials (aniline and AgNO_3_) was 2:1, the initial specific capacity of poly (AN–Ag–S) composite cell was the highest: 1114 mAh/g. After 100 cycles, the specific capacity was kept at 573 mAh/g. Therefore, the introduction of Ag into the composite cathode material can effectively enhance the conductivity and improve the high-rate performance of the battery.

## 2. Experiment

### 2.1. Material Preparation

A certain quantity of aniline (AN, AR, Ji’nan Tian Shi Hao Trading Co., Ltd.; Jinan, China) was put into 2.5 mM sodium dialkylsulfonate (SDS, AR, Beijing Yili Fine Chemicals Co., Ltd.; Beijing, China) solvent. The mixture was stirred for 30 min and then ammonium persulfate (APS, AR, Beijing Yili Fine Chemicals Co., Ltd.; Beijing, China) was dropped into the mixture slowly. Silver nitrate (AgNO_3_, AR, Beijing baileibo Technology Co., Ltd.; Beijing, China) (separately 1:2 wt, 1:4 wt, 1:8 wt feed ratios with AN) was subsequently dropped into the mixture. After being stirred for 24 h at room temperature, the final precipitate was washed, dried and ground. Then, the product powder was blended evenly with sulfur (S, AR, Beijing Yili Fine Chemicals Co., Ltd.; Beijing, China) (1:2 wt), and heated for 24 h at 160 °C.

### 2.2. Characterization

In order to determine the elemental composition, the polyaniline–silver–sulfur (poly (AN–Ag–S)) composites were characterized via infrared spectroscopy (IR, Nicolet-60SXB; Thermo Nicolet, Waltham, MA, USA), X-ray Diffraction (XRD, Rigaku D/Max 2550; Rigaku International Corporation, Japan) and Raman spectra (LabRAM HR800; Horiba Jobin Yvon, France). With the heating rate of 10 °C/min, thermogravimetric analysis (TGA, TA-Q50; New Castle, DE, USA) was used to confirm the component of the product. Moreover, the surface topography and particle size of the composite have been observed via scanning electron microscopy (Zooma 200; Eindhoven, The Netherlands).

### 2.3. Electrochemical Measurement

The binder (polyvinylidene fluoride, PVDF, SOLVAY-SOLEF-9007, Qingdao, China) was blended with poly (AN–Ag–S) composites at the mass ratio of 1:8. Meanwhile, the ethanol absolute (AR, Hubei xinrunde Chemical Co., Ltd.; Hubei, China) and deionized water were put into the mixture. After being heated in a water bath for 8 h, the mixture became viscous. Then, the applicator blade was used to apply the slurry uniformly onto an aluminum foil current collector, which was subsequently dried in a vacuum drying oven (DZF 2001 type, Shanghai Yiheng Instrument Co., Ltd.; Shanghai, China) at 60 °C for 24 h. After that, it was punched into 1 cm-diameter circular electrode pieces. These punched pieces were used as a positive electrode, the lithium piece (15.6 × 0.45 mm, 16 × 0.6 mm, Jiangsu Taizhou) was used as a negative electrode, the Celgard 2400 microporous film was used as the separator and 1 mol/L of LiTFSI (1,3-dioxolane, DOL/dimethoxyethane, DME; volume ratio of 1:1) was used as the electrolyte. Button battery samples were later assembled in the glove box (Lab 2000) under vacuum conditions. After standing still after 24 h, the samples were electrochemically tested. With the specific capacity and current multiplying ratio calculated on the mass of the active substance sulfur, the constant current charge and discharge testing of this composite positive electrode was carried out in the LAND CT 2001 A Blue Test System (Wuhan, China).

## 3. Results and Discussion

### 3.1. Characterization of Poly (AN–Ag–S) Composites

**Figure 1:**
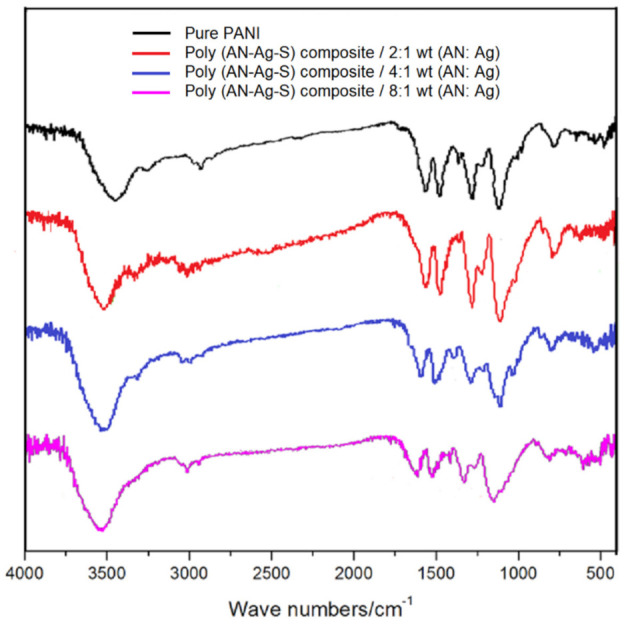
The Infrared Spectrum of pure PANI and poly (AN–Ag–S) composites with different Ag contents.

The infrared spectrum (Figure 1) clearly showed that the curves of poly (AN–Ag–S) composites with different Ag contents were similar with the curve of pure PANI. The signal at 3500 cm^−1^ showed the existence of amines and imines. The signals at 1570 cm^−1^ and 1505 cm^−1^ were caused by the vibrations of quinoid N=Q=N and C=C bonds in the benzene ring; the signal at 1310 cm^−1^ was caused by the vibration of the C–N bond and the signal at 1130 cm^−1^ was attributed to the in-plane bending of the C–H bond in the benzene ring, while the signal at 880 cm^−1^ was attributed to the out-of-plane bending of the C–H bond in the benzene ring [11]. Compared with the IR curve of pure PANI, the peaks in the IR curves of poly (AN–Ag–S) composites were displaced, because the existence of Ag decreased the electron cloud density of PANI molecular chains and caused the shift of main characteristic peaks [12,13,14]. Moreover, when the Ag contents changed, the strength and the location of the main characteristic peaks also changed in the IR curves of poly (AN–Ag–S) composites.

**Figure 2:**
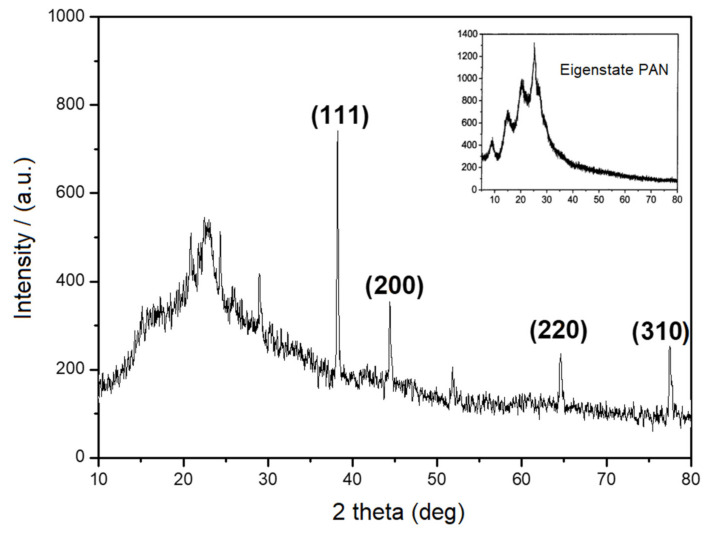
XRD of poly (AN–Ag–S) composite.

**Figure 3:**
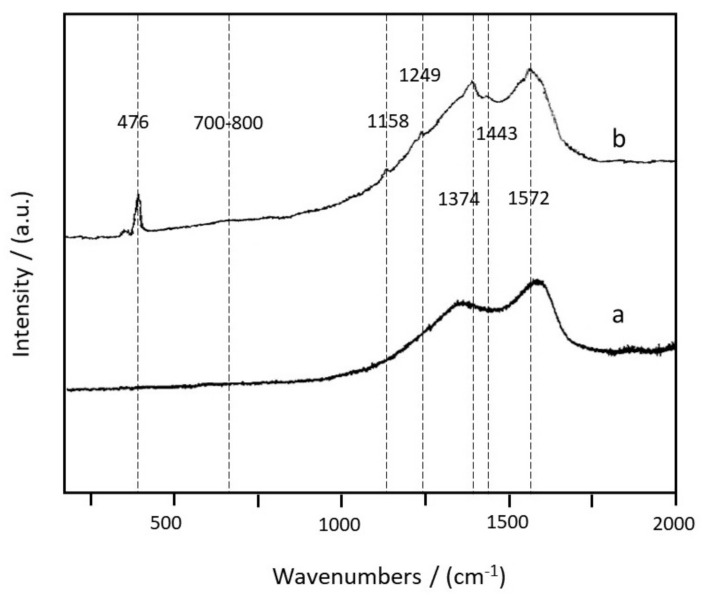
(**a**) Raman spectra of pure PANI; (**b**) Raman spectra of poly (AN–Ag–S) composite.

To further demonstrate the elemental composition, XRD characterization was conducted. In Figure 2, according to JCPDS NO.4-0783, the signals at 38.1°, 44.5°, 64.7° and 77.8° were assigned to (111), (200), (220) and (311) lattice planes of Ag [15], indicating the existence of the face-centered cubic structure. Compared with the XRD of eigenstate PANI, the intensity of diffraction peak in XRD of the poly (AN–Ag–S) composite decreased, which was due to the interaction between Ag and PANI. The signals at 23.3° and 28.1° were assigned to the element sulfur, which demonstrated that sulfur maintained its original crystal structure in the poly (AN–Ag–S) composite [16]. Raman spectra of pure PANI and poly (AN–Ag–S) composites can be seen in Figure 3. In the curve (a) of pure PANI, the characteristic peak around 1374 cm^−1^ was caused by the vibration of polaron C-N^+^, while the characteristic peak around 1572 cm^−1^ was due to the stretching vibration of C=C in the quinone ring. In the curve (b) of the poly (AN–Ag–S) composite, the peaks in 700–800 cm^−1^ were attributed to the deformation of imine in the quinone ring, which might be the chemical bonds among silver nanoparticles and imino groups in the polyaniline quinone ring. The peak at 1158 cm^−1^ was attributed to the bending vibration of C–H in the quinone ring, the peak at 1249 cm^−1^ was attributed to the stretching of C–N in the benzene ring, the peak at 1443 cm^−1^ was attributed to the stretching of C–C in the quinone ring and the peak at 476 cm^−1^ was characteristic of sulfur [17], which demonstrated that sulfur was successfully added into the poly (AN–Ag–S) composite.

**Figure 4:**
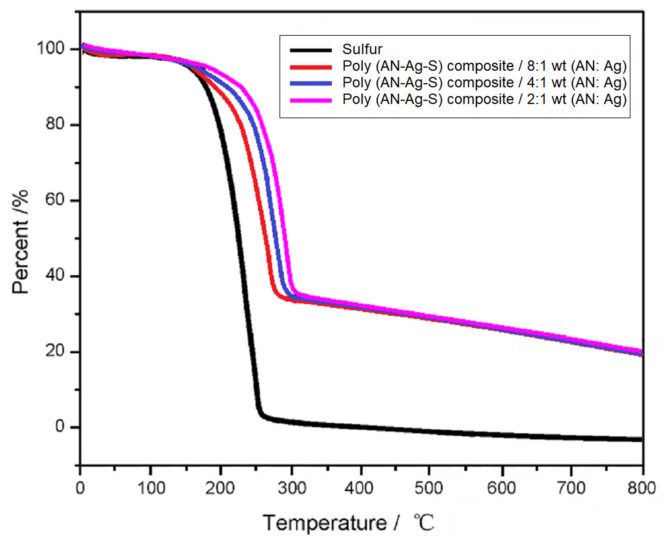
The TGA curves of poly (AN–Ag–S) composites with different Ag contents.

In Figure 4, the weight loss curve of poly (AN–Ag–S) composites was plotted against the reference curve of elemental sulfur. Its decomposition step started at 16°C, which was attributed to the loss of sulfur. The weight loss of poly (AN–Ag–S) composite was about 62 wt%, because parts of elemental sulfur were lost during the preparation process. With the increase in Ag contents, the decomposition temperature of the poly (AN–Ag–S) composite increased, which indicated that the existence of Ag particles can help to enhance the thermal stability of the composites [18].

**Figure 5:**
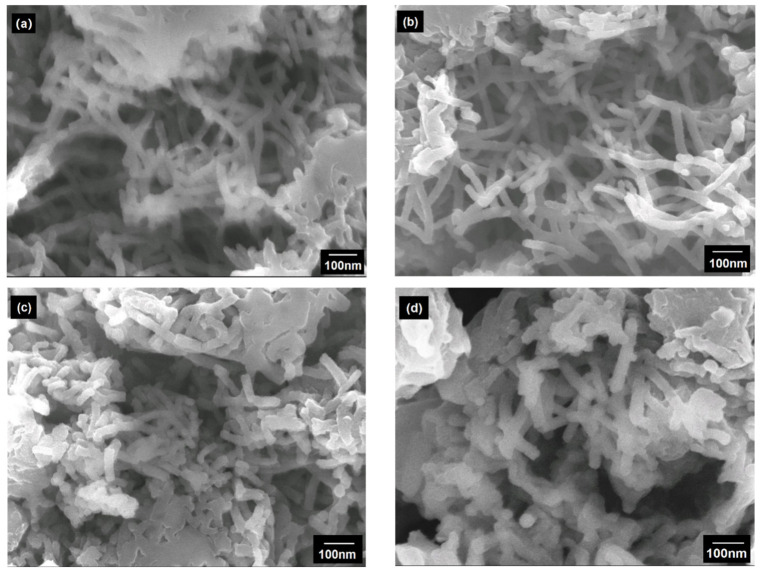
(**a**) Scanning electron microscope of poly (AN–Ag) composite/2:1 wt (AN: Ag); (**b**) scanning electron microscope of poly (AN–Ag) composite/4:1 wt (AN: Ag); (**c**) scanning electron microscope of poly (AN–Ag) composite/8:1 wt (AN: Ag); (**d**) scanning electron microscope of poly (AN–Ag–S) composite.

To further demonstrate the structure of poly (AN–Ag–S) composites, SEM was utilized to investigate the morphologies. In Figure 5a–c, the poly (AN-Ag) composites with different Ag contents all presented nanotube structures. It can be inferred that SDS acted as the micelle template, providing “cavity” in a dynamic equilibrium; therefore, the aniline monomer can diffuse through the “cavity” wall [19,20]. Then, aniline monomers and generated Ag particles assembled along the SDS micelles, producing tube nano composite. However, with the increase in Ag contents in the composites, the agglomeration became worse; because there was no stabilizer and the stirring rate became relatively low during the synthesis process, thus the polymerization rate of Ag^+^ was faster than that of aniline monomers, which caused the clustering of Ag particles, and then PANI was polymerized on the surface of Ag particles. Therefore, when the content of Ag was lower, the chains of polymerized PANI can be fully stretched, forming a potential resistance layer, which would prohibit the further agglomeration [21,22]. After the poly (AN–Ag) composite had been heated with sulfur, the morphology of the product rarely changed (shown in Figure 5d).

### 3.2. Electrical Measurement

**Figure 6:**
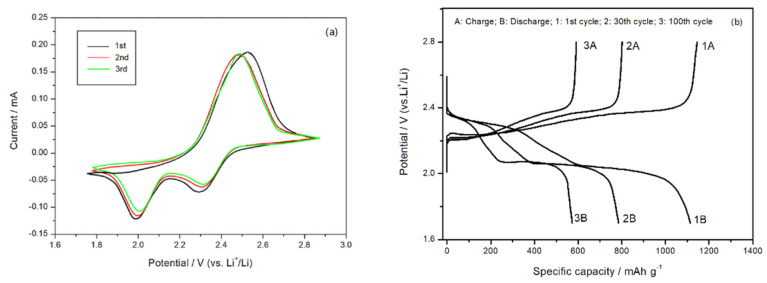
(**a**) CV curves of poly (AN–Ag–S) composite cathode material; (**b**) the charge–discharge curves of poly (AN–Ag–S) composite.

When employed as the active cathode materials in a Li–S battery, the electrochemical properties of poly (AN–Ag–S) composites were tested. Figure 6a depicted the cyclic voltammetry (CV). The concentration of the LiTFSI/DOL-DME electrolyte was 1 M; the scan rate was set at 0.05 mV/s, and the voltage range was set at 1.2–3.0 V. During the first cycle, two distinct reduction peaks appeared at 2.3 V and 2.0 V; one peak referred to the conversion of elemental sulfur S_8_ changing to the high-valence polysulfide S^2−^_x_ (x = 4 − 6), and the other corresponded with the reduction of high-valence polysulfide S^2−^_x_ to Li_2_S_2_, Li_2_S, etc. [23,24,25]. During the subsequent forward scan, a distinct oxidation peak appeared at 2.45 V. It meant the conversion of Li_2_S to polysulfide and then to elemental sulfur S_8_. From Figure 6, the CV curve of the second cycle coincided with the one of the third cycle, indicating the existence of good cycle stability [26]. When the open-circuit voltage was set to 2.8 V and the current was set to 0.1 C, two obvious plateaus (Figure 6b) appeared at 2.33 V and 2. 05 V in the charge–discharge curve of the poly (AN–Ag) composite cathode material, which was characteristic of liquid electrolyte Li–S batteries [27].

**Figure 7:**
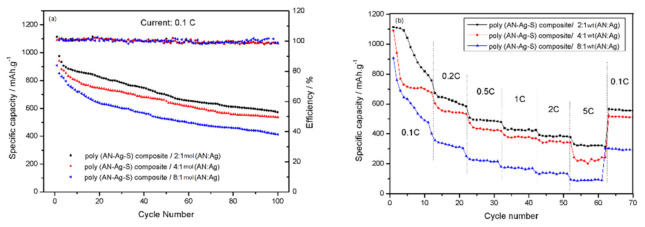
(**a**) The cycle performance and coulombic efficiency curves of poly (AN–Ag–S) composite/8:1 wt (AN: Ag), poly (AN–Ag–S) composite/4:1 wt (AN: Ag) and poly (AN–Ag–S) composite/2:1 wt (AN: Ag); (**b**) the rate performance curves of the poly (AN–Ag–S) composite/8:1 wt (AN: Ag), poly (AN–Ag–S) composite/4:1 wt (AN: Ag) and poly (AN–Ag–S) composite/2:1 wt (AN: Ag).

In Figure 7a, the initial specific capacity of poly (AN–Ag–S) composite/8:1 wt (AN: Ag) electrode was 907.88 mAh/g. During the first 40 cycles, the specific capacity sharply decreased to 567 mAh/g. Then, the specific capacity became stable, with the specific capacity of 412 mAh/g, the capacity retention rate around 45.4% and the coulombic efficiency of 98% after 100 cycles. Comparatively, the initial specific capacity of poly (AN–Ag–S) composite/4:1 wt (AN: Ag) electrode was 1091 mAh/g, and the discharge specific capacity reduced to 681 mAh/g after 40 cycles, finally to 536 mAh/g after 100 cycles, with the capacity retention rate around 49.1% and the coulombic efficiency of 98%, which showed a better cycle stability. Moreover, the initial specific capacity of poly (AN–Ag–S) composite/2:1 wt (AN: Ag) electrode increased to 1114 mAh/g, and the discharge-specific capacity can keep above 573 mAh/g after 100 cycles, with the capacity retention rate around 51.4% and the coulombic efficiency of 98%. It means that this electrode owned the best cycle stability. It can be inferred that, when PANI compounded with Ag, the polar groups in PANI structure might be bonded with Ag particles, which made the PANI chains stretch larger and increased the conjugation degree of PANI [28,29,30]. So, it became easy for the charges to transfer and the electrical conductivity of the enhanced composite [31,32]. Additionally, when the Ag content was higher, the layer of PANI–Ag might be more continuous, which might form a better conductive circuit.

When the current density was set to different levels, such as 0.1 C, 0.2 C, 0.5 C, 1 C, 2 C, 5 C, in the charge–discharge process (10 times/each current density), the rate performance can be seen in Figure 7b. The discharge-specific capacities of poly (AN–Ag–S) composite/8:1 wt (AN: Ag) electrode were about 908 mAh/g, 401 mAh/g, 252 mAh/g, 180 mAh/g, 142 mAh/g and 95 mAh/g. When the current density was readjusted to 0.1 C, the discharge-specific capacity of the battery returned to 303 mAh/g. Comparatively, the discharge-specific capacities of the poly (AN–Ag–S) composite/4:1 wt (AN: Ag) electrode were 1091 mAh/g, 603 mAh/g, 473 mAh/g, 392 mAh/g, 358 mAh/g and 243 mAh/g, separately. The discharge-specific capacity returned to 519 mAh/g. The discharge-specific capacities of poly (AN–Ag–S) composite/2:1 wt (AN: Ag) electrode were 1114 mAh/g, 673 mAh/g, 507 mAh/g, 439 mAh/g, 392 mAh/g and 331 mAh/g, with the discharge-specific capacity of 567 mAh/g when the current was readjusted to 0.1 C. It was obvious that, when the Ag contents increased, the composite had a superior high-rate performance. It also confirmed that the poly (AN–Ag–S)/2:1 wt (AN: Ag) composite had better electrical conductivity.

## 4. Conclusions

In this experiment, a new kind of polyaniline–silver–sulfur nanotube composite was successfully synthesized and characterized. With the increase in Ag contents in the composite, the thermal stability of the composite enhanced. When the feed ratio of AN and AgNO_3_ was 2:1, the poly (AN–Ag–S) composite electrode owned the highest initial specific capacity of 1114 mAh/g, the best cycle performance, the best capacity retention abilities (573 mAh/g after 100 cycles) and superior high-rate performance. The introduction of Ag into composite cathode material can effectively increase the electrochemical activity of the electrode, solve the problem of the poor conductivity in sulfur and improve the battery performance.

## Data Availability

Data sharing is not applicable for this article.

## References

[1] Zou M., Yoshio M., Gopukumar S., Yamaki J.-I. (2003). Synthesis of High-Voltage (4.5 V) Cycling Doped LiCoO2 for Use in Lithium Rechargeable Cells. Chem. Mater..

[2] Pradhan N., Pal A., Pal T. (2002). Silver nanoparticle catalyzed reduction of aromatic nitro compounds. Colloids Surf. A Physicochem. Eng. Asp..

[3] Saikat M., Debabrata R., Murali S. (2003). Ag^+^-Keggin ion colloidal particles as novel templates for the growth of silver nanoparticle assemblies. J. Mater. Chem..

[4] Hsinglin W., Wenguang L., Jia Q.X., Akhadov E. (2007). Tailoring conducting polymer chemistry for the chemical deposition of metal particles and clusters. Chem Mater..

[5] Bober P., Stejskal J., Mrchová T., Prokeš J. (2011). Polyaniline–silver composites prepared by the oxidation of aniline with mixed oxidants, silver nitrate and ammonium peroxydisulfate: The control of silver content. Polymer.

[6] Tahmina N., Aamir R., Sara A., Sarwar N. (2021). A facile approach to synthesize ZnO-decorated titanium carbide nanoarchitectures to boost up the photodegradation performance. Ceram. Int..

[7] Nasir S., Seung H.C., Ghulam D., BinHumayoun U., Kumar M., Nawaz A., InJeong D., AlamZaidI S.F., HoYoon D. (2021). Synthesis of citrate-capped copper nanoparticles: A low temperature sintering approach for the fabrication of oxidation stable flexible conductive film. Appl. Surf. Sci..

[8] And Y.S., Xia Y. (2002). Large-scale synthesis of uniform silver nanowires through a soft, self-seeding, polyol process. Adv. Mater..

[9] Ki-Seok K., Soo-Jin P. (2012). Bridge effect of silver nanoparticles on electrochemical performance of graphite nanofiber/polyaniline for supercapacitor. Synthetic Met..

[10] Sawangphruk M., Kaewsongpol T. (2012). Direct electrodeposition and superior pseudocapacitive property of ultrahigh porous silver-incorporated polyaniline films. Mater. Lett..

[11] Zhongbao W., Shichao Z., Lan Z., Lin R., Wu X., Fang H., Ren Y. (2014). Hollow spherical carbonized polypyrrole/sulfur composite cathode materials for lithium/sulfur cells with long cycle life. J. Power Sources.

[12] Yakuphanoglu F., Basaran E., Şenkal B.F., Sezer E. (2006). Electrical and Optical Properties of an Organic Semiconductor Based on Polyaniline Prepared by Emulsion Polymerization and Fabrication of Ag/Polyaniline/n-Si Schottky Diode. J. Phys. Chem. B.

[13] Suber L., Sondi I., Matijević E., Goia D.V. (2005). Preparation and the mechanisms of formation of silver particles of different morphologies in homogeneous solutions. J. Colloid Interface Sci..

[14] Shaolin M., Jingqing K. (1998). Energy density and IR spectra of polyaniline synthesized electrochemically in the solutions of strong acids. Synthetic Met..

[15] Stejskal J., Prokeš J., Trchová M. (2008). Reprotonation of polyaniline: A route to various conducting polymer materials. React. Funct. Polym..

[16] Khanna P., Singh N., Charan S., Viswanath A.K. (2005). Synthesis of Ag/polyaniline nanocomposite via an in situ photo-redox mechanism. Mater. Chem. Phys..

[17] Xuewu G., Yonghong N., Ye Q. (2001). Novel irradiation template approach to prepare silver nano-ribbons. J. Radiat. Res. Radiat. Process.

[18] Rowan S.J., Cantrill S.J., Cousins G.R.L., Sanders J.K.M., Stoddart J.F. (2002). Dynamic covalent chemistry. Angew. Chem. 1nt. Ed..

[19] Wu D., Fang Y. (2004). Surface-enhanced Raman scattering of a series of n-hydroxybenzoic acids (n = P, M and O) on the silver nano-particles. Spectrochim. Acta Part A Mol. Biomol. Spectrosc..

[20] Mbhele Z.H., Salemane M.G., van Sittert C.G.C.E., Nedeljkovic J., Djokovic V., Luyt A. (2003). Fabrication and characterization of silver polyvinyl alcohol nanocomposites. Mater. Chem. Phys..

[21] Wang J., Zhang S. (2020). Synthesis and properties of polyaniline-sulfur with different nanostructures: Via interfacial emulsification method and micelle template method. RSC Adv..

[22] Jing W., Shichao Z. (2020). Serial disulfide polymers as cathode materials in lithium-sulfur battery materials optimization and electrochemical characterization. Appl. Sci..

[23] Guohui C., Yonglan L., Wenbo L., Qin X., Asiri A.M., Al-Youbi A.Q., Sun X. (2012). Ag nanoparticles decorated polyaniline nanofibers: Synthesis, characterization, and applications toward catalytic reduction of 4-nitrophenol and electrochemical detection of H_2_O_2_ and glucose. Catal. Sci. Technol..

[24] Barros R., Azevedo W.M.D. (2008). Polyaniline/silver nanocomposite preparation under extreme or non-classical conditions. Synth. Met..

[25] Park S., An J., Potts J.R., Velamakanni A., Murali S., Ruoff R.S. (2011). Hydrazine-reduction of graphite- and graphene oxide. Carbon.

[26] Hou T., Xu W.-T., Chen X., Peng H., Huang J.-Q., Zhang Q. (2017). Lithium Bond Chemistry in Lithium-Sulfur Batteries. Angew. Chem. Int. Ed..

[27] Bourrat X. (1993). Electrically conductive grades of carbon black: Structure and properties. Carbon.

[28] Rauh R.D., Abraham K.M., Pearson G.F., Surprenant J.K., Brummer S.B. (1979). A lithium/dissolved sulfur battery with an organic electrolyte. J. Electrochem. Soc..

[29] Li W., Zhang Q., Zheng G., Seh Z.W., Yao H., Cui Y. (2013). Understanding the Role of Different Conductive Polymers in Improving the Nanostructured Sulfur Cathode Performance. Nano Lett..

[30] López-Palacios J., Muñoz E., Heras M.A., Colina A., Ruiz V. (2006). Study of polyaniline films degradation by thin-layer bidimensional spectro electrochemistry. Electrochim. Acta..

[31] Prasad K.R., Munichandraiah N. (2002). Electrooxidation of methanol on polyaniline without dispersed catalyst particles. J. Power Sources.

[32] Dominis A.J., Spinks G.M., Kane-Maguire L.A., Wallace G.G. (2002). A de-doping/re-doping study of organic soluble polyaniline. Synth. Met..

